# Reply to: Food insecurity and mortality in older adults: a prospective reconstruction

**DOI:** 10.1007/s40520-026-03506-0

**Published:** 2026-09-08

**Authors:** Vincenza Gianfredi, Daniele Nucci, Pinar Soysal, Stefania Maggi, Alberto Castagna, Andrea Cozza, Vincenzo Baldo, Nicola Veronese

**Affiliations:** 1 Department of Cardiac, Thoracic, Vascular Sciences, and Public Health, University of Padua, via Loredan 18, Padua, 35122 Italy; 2 Struttura Semplice Dipartimentale Igiene Alimenti e Nutrizione, Dipartimento di Igiene e Prevenzione Sanitaria, Agenzia di Tutela della Salute (ATS) Brescia, Brescia, Italy; 3 PhD National Program in One Health Approaches to Infectious Diseases and Life Science Research, Department of Public Health, Experimental and Forensic Medicine, University of Pavia, Pavia, Italy; 4 Department of Geriatric Medicine, Faculty of Medicine, Bezmialem Vakif University, Istanbul, Turkey; 5 National Research Council (CNR), Aging Section, Padua, Italy; 6 Department of Primary Care, Health District of Soverato, Azienda Sanitaria Provinciale Catanzaro, Soverato, Italy; 7 Faculty of Medicine, Saint Camillus International University of Health Sciences, Rome, Italy

To the Editor,

We thank Tursunov and colleagues for their thoughtful comments on our longitudinal analysis of food insecurity and mortality in the English Longitudinal Study of Ageing (ELSA) [1]. Their letter identifies valuable priorities for future research, particularly the use of causal diagrams, repeated nutritional assessment, and alternative measures of early mortality. However, several criticisms appear to treat acknowledged limitations as if they invalidated the observed association, and they repeatedly frame our findings as a causal claim. Our study was designed to estimate a prospective association and to evaluate food insecurity as a predictor of mortality, not to establish that food insecurity was a proven cause of death. We explicitly stated that observational data do not permit definitive causal inference, that some adjusted covariates could be mediators, and that nutritional mechanisms were indirectly inferred rather than directly measured [1]. This distinction is central to the interpretation of our results.

## Premature mortality as a standardized outcome

We agree that no single age threshold fully captures the individual experience of early death. Nevertheless, death before age 75 is not merely a convenient or idiosyncratic definition. The under-75 threshold is embedded in English public-health surveillance for several premature-mortality indicators [2], and it has also been used as the endpoint for premature all-cause and cardiovascular mortality in recent large, nationally representative cohort analyses [3]. Fixed thresholds are intended to provide a reproducible population-level metric; they are not individualized estimates of remaining life expectancy.

The proposal to redefine premature death according to sex- or deprivation-specific life expectancy raises a different problem. Lower life expectancy in disadvantaged groups is itself an expression of health inequality. Using a lower threshold for those groups could classify deaths that are premature from an equity and prevention perspective as “expected,” thereby normalizing rather than identifying disparities. Area-level life expectancy also cannot be assumed to represent an individual counterfactual lifespan.

The comparison with Ma et al. does not show that our estimate is an artifact of the cut-off [4]. Ma et al. defined premature mortality as death before age 80, but their US cohort differed from ELSA in age structure, exposure instrument, food-insecurity prevalence, follow-up, social context, and covariate specification. Differences between hazard ratios cannot therefore be attributed to the age threshold alone. Importantly, Men et al. observed a graded association between household food insecurity and premature mortality in more than 500,000 Canadian adults [5], while Tian et al. reported higher under-75 mortality across 10-, 6-, and 2-item food-security instruments [3].

Our models adjusted for baseline age as a continuous variable. This is especially relevant because food-insecure participants were substantially younger than food-secure participants in ELSA. Since age strongly predicts mortality, this imbalance can suppress the crude association and helps explain why adjustment strengthened the estimate for premature mortality. We agree that sensitivity analyses using alternative thresholds, attained age as the analysis time scale, or years of potential life lost would add useful information. Their absence is a limitation, but it does not render the standardized under-75 endpoint biologically meaningless or methodologically invalid.

## Nutritional status and proposed mechanisms

Tursunov et al. correctly note that food insecurity and malnutrition are not interchangeable. This distinction was already made in our article: the ELSA item captured meal reduction or omission because of insufficient money and did not directly measure dietary quality, nutritional adequacy, or malnutrition [1]. We described nutritional mechanisms as plausible and indirectly supported, not as a mediation pathway demonstrated within our dataset. Measurement of every intermediate mechanism is not required to estimate the total prospective association between an exposure and an outcome; it is required when the objective is to quantify mediation.

The external evidence cited in the correspondence largely supports, rather than refutes, the plausibility of the pathway. In a nationally representative longitudinal study of Mexican adults aged 50 years or older, moderate and severe food insecurity were associated with increasing rates of sarcopenia, and incident severe food insecurity predicted the cumulative incidence of sarcopenia and severe sarcopenia [6]. In the Hong Kong study cited by Tursunov et al., food insecurity was associated with malnutrition risk, possible sarcopenia, falls, and frailty; malnutrition risk mediated part of these associations [7]. Because that study was cross-sectional, its mediation estimates cannot establish temporal causality, but they support the presence of a nutritional pathway. Earlier work using the Mini Nutritional Assessment likewise found food insecurity to be independently associated with malnutrition risk among community-dwelling older adults [8].

Food insecurity can coexist with obesity because sufficient or excessive energy intake does not guarantee adequate protein, micronutrient intake, or diet quality. Therefore, the existence of energy-dense dietary patterns among food-insecure adults does not negate the possibility of nutritional vulnerability or sarcopenia. We agree that future ELSA analyses incorporating validated nutritional instruments and objective measures of muscle health would permit direct testing of these mechanisms. The lack of such measures limits mechanistic interpretation, but it does not invalidate the mortality association.

## Confounders, mediators, and the change after adjustment

We also agree that directed acyclic graphs can improve covariate selection [9]. However, multimorbidity, obesity, disability, and physical inactivity cannot be classified as mediators solely because they may occur downstream of food insecurity. In older adults, these conditions can precede food insecurity and increase its probability through health-related expenditures, reduced earnings, mobility restrictions, difficulty shopping or cooking, and greater dependence on others. Their role is therefore potentially bidirectional and depends on the causal question, timing, and assumed data-generating structure.

It is also important to distinguish overadjustment from collider bias. Adjustment for a true mediator blocks part of the pathway when the estimand is the total effect and may produce overadjustment bias [10]. Collider bias, by contrast, requires conditioning on a variable caused by two or more variables in the relevant causal graph; a mediator is not automatically a collider [9]. Our article explicitly acknowledged that the fully adjusted model estimates an association conditional on the included health and lifestyle factors rather than a total causal effect [1].

Most importantly, an increase in the hazard ratio after adjustment is not diagnostic of collider stratification. Estimates may move away from the null because of negative confounding or suppression. In our cohort, food-insecure participants were on average 4.6 years younger and were less likely to report ever smoking than food-secure participants. Both imbalances could mask rather than generate a positive mortality association in the crude model. Thus, the change from the crude to the adjusted estimate has a coherent epidemiological explanation and cannot, by itself, identify collider bias. A sequential set of models separating sociodemographic factors from potentially intermediate health variables would improve transparency in future analyses; nevertheless, the crude and fully adjusted estimates already show that the result is sensitive to the covariate structure, a point we reported and discussed.

## Diet quality and the totality of evidence

We agree that diet quality is an important potential mediator. However, the findings of Beydoun et al. should not be interpreted as demonstrating that food insecurity is unrelated to mortality [11]. That study of 2,894 older US adults reported inconsistent findings for food insecurity: no association in the reduced model and an inverse association in the fully adjusted model. Its authors did not conclude that food insecurity has no mortality relevance; rather, they called for further longitudinal research. The study also involved a markedly older analytic sample, a different food-security instrument, substantial exclusions needed to assemble the diet-dementia sample, and a different follow-up period. It is therefore informative but not directly dispositive for ELSA.

More broadly, the available evidence does not rest on a single cohort. A 2025 meta-analysis of 19 cohort studies found food insecurity to be associated with higher mortality overall (pooled HR 1.23), with a severity gradient and a pooled HR of 1.52 for severe food insecurity [12]. Large studies in Canada and the United States have also reported graded or significant associations with all-cause, premature, and cardiovascular mortality [4, 5, 13, 14]. Tian et al. further showed that low diet quality and other cardiometabolic factors may attenuate the association when added as potential intermediates [3]. This finding is compatible with mediation; it does not imply that food insecurity is irrelevant. Indeed, requiring diet quality to be included in a model estimating the total association would conflict with the warning against adjustment for mediators.

Food insecurity may affect survival through several pathways beyond diet composition, including psychosocial stress, depression, competing expenditure on food and medicines, difficulties accessing health care, social isolation, and exacerbation of chronic disease. A formal mediation analysis could distinguish these pathways, but it would require temporal ordering and strong assumptions regarding exposure-mediator, mediator-outcome, and exposure-outcome confounding.

In conclusion, we share the call for analyses using alternative premature-mortality metrics, causal diagrams, repeated food-security assessment, nutritional screening, and validated diet-quality indices. These additions would refine the estimand and clarify mechanisms. They do not, however, overturn the central finding of our study: a severe, financially driven reduction in food intake identified a small but vulnerable group of ELSA participants with a higher subsequent mortality hazard. This result should be interpreted as a longitudinal association and a prognostic signal, not as definitive proof of causality. Its consistency with several large cohorts and a recent meta-analysis supports continued investigation and the clinical and public-health relevance of identifying food insecurity among older adults.

## Data Availability

No new data were generated or analyzed for this response.
